# Individualized active surveillance for carbapenem-resistant microorganisms using Xpert Carba-R in intensive care units

**DOI:** 10.1038/s41598-023-36321-y

**Published:** 2023-06-12

**Authors:** Shuliang Zhou, Sulin Mi, Xin Rao, Qi Zhang, Shiwen Wei, Meng Xiao, Zhiyong Peng, Jing Wang

**Affiliations:** 1 Department of Critical Care Medicine, Zhongnan Hospital of Wuhan University, Donghu Road 169, Wuhan, 430071 Hubei China; 2 Clinical Research Center of Hubei Critical Care Medicine, Donghu Road 169, Wuhan, 430071 Hubei China; 3 Department of Cardiovascular Ultrasound, Zhongnan Hospital of Wuhan University, Wuhan University, Wuhan, 430071 China

**Keywords:** Microbiology, Medical research

## Abstract

Carbapenem antibiotics are widely used in ICU, and the prevalence of carbapenem-resistant microorganisms (CRO) has increased. This study aimed to assess the role of individualized active surveillance using Xpert Carba-R of carbapenem resistance genes on CRO risk. A total of 3,765 patients were admitted to the ICU of Zhongnan Hospital of Wuhan University between 2020 and 2022. The presence of carbapenem resistance genes were monitored using Xpert Carba-R, and CRO incidence was assigned as the investigated outcome. Of 3,765 patients, 390 manifested the presence of CRO, representing a prevalence of 10.36%. Active surveillance using Xpert Carba-R was associated with a lower CRO risk (odds ratio [OR]: 0.77; 95% confidence interval [CI] 0.62–0.95; *P* = 0.013), especially for carbapenem-resistant Acinetobacter + carbapenem-resistant *Pseudomonas aeruginosa* (OR: 0.79; 95% CI 0.62–0.99; *P* = 0.043), carbapenem-resistant *Klebsiella pneumoniae* (OR: 0.56; 95% CI 0.40–0.79; *P* = 0.001), and carbapenem-resistant Enterobacteriaceae (OR: 0.65; 95% CI 0.47–0.90; *P* = 0.008). Individualized active surveillance using Xpert Carba-R may be associated with a reduction in the overall CRO incidence in ICU. Further prospective studies should be performed to verify these conclusions and guide further management of patients in ICU.

## Introduction

Antibiotic resistance is a global threat to health care and is associated with complex and expensive treatments, longer hospital stays, and high mortality^1^. Multiple antibiotic resistance in gram-negative bacilli, including *Klebsiella pneumoniae*, *Acinetobacter baumannii*, and *Pseudomonas aeruginosa*, has dramatically increased over the last few years, thus threatening human health^2^. Carbapenem-resistant microorganisms (CRO) are associated with prolonged hospitalization and, owing to their rapid spread worldwide, contribute to an urgent public health threat^3^. Treatment and prevention of CRO are difficult owing to its fast transmission speed, wide range, and high fatality rate^4–6^.

Patients in intensive care units (ICU) are at high risk of CRO colonization and subsequent CRO infection. Thus, early detection, isolation, and treatment of CRO are important for the treatment of critical illnesses. Guidelines for the prevention and control of carbapenem-resistant Enterobacteriaceae (CRE), carbapenem-resistant Acinetobacter (CRAs), and carbapenem-resistant *P. aeruginosa* (CRPA) in healthcare facilities indicate that patients should be actively screened when they enter the hospital for treatment or after exposure to risk to avoid delayed treatment^7^. Active surveillance of CRO infection and surveillance cultures of asymptomatic CRO colonization allows the early introduction of infection prevention and control measures to prevent transmission to other patients and the environment^8–11^. Studies have demonstrated that patients with CRO colonization have an approximately 1.79-fold greater risk of dying in the ICU than non-colonized patients, and CRO colonization is associated with longer hospital stays and substantial health care costs^7,8,12^.

Routine active surveillance involves rectal cultures using dried rayon swabs, while routine methods with lower diagnostic performance and longer examination duration take a long time. Currently, Xpert Carba-R is a useful tool for the rapid and accurate detection of patients carrying potentially multidrug-resistant bacteria^13,14^. The addition of oxacillinase (OXA)-181 and OXA-232 carbapenem resistance genes to the panel detected using Xpert Carba-R could enable more rapid detection as well as identify the five most popular carbapenem antimicrobial resistance mechanisms: *K.* *pneumoniae* carbapenemase (KPC), New Delhi metallo-β-lactamase (NDM), Verona integron-mediated metallo-β-lactamase (VIM), imipenemase (IMP)-1, and OXA-48. The Xpert Carba-R reaction box can rapidly detect 91 resistant genes from five different gene families within 1 h, which could prevent unnecessary isolation and reduce the risk of nosocomial cross-infection. In this study, we retrospectively analyzed CRO surveillance data in the ICU from 2020 to 2021, and active screened the high-risk patients by Xpert Carba-R and analyzed all CRO surveillance data from 2021 to 2022, and then assessed the role of individualized active surveillance of carbapenem resistance genes on CRO risk.

## Methods

### Study design and population

This single-center, before-after study was approved by the ethics committee of Zhongnan Hospital of Wuhan University (no: 2021023). From 2020 to 2022, all patients admitted to the ICU will be screened for CRO when necessary (such as infection is suspected) by traditional laboratory culture. Prior to 2021, CRO surveillance is mainly obtained by routine specimen culture rather than by Xpert Cabar-R; since 2021, active surveillance for CRO by Xpert Carba-R was added to our cluster infection control measures. In the first period, we retrospectively studied the clinical data of all patients admitted to the ICU of Zhongnan Hospital from March 2020 to March 2021 as the control group. In the second period, active surveillance of high-risk patients using Xpert Carba-R and routinely culture of microorganisms were implemented from April 2021 to April 2022 (Supplementary S1). We evaluated the effectiveness of individualized active surveillance of carbapenem resistance genes using Xpert Carba-R in relation to CRO incidence. Inclusion criteria for patients at high risk for CRO were as follows: (1) individuals who did not respond to conventional anti-infective therapy; (2) patients transferred from long-term care facilities or other hospitals; (3) critically ill patients who were transferred from the general ward with a hospital stay of more than 3 days; (4) patients with fecal incontinence; and (5) patients treated with transplant, long-term immunosuppressant use, or chemotherapy. We have obtained informed consent from all patients or their legal guardians. All methods were performed in accordance with relevant guidelines and regulations.

### Active surveillance by Xpert Carba-R between 2021 and 2022

After inclusion, high-risk patients immediately underwent routine rectal swabs by trained nurses. If surveillance results were negative, rectal swabs were monitored regularly once a week. If an infection was suspected during hospitalization, active surveillance was performed at any time. If Xpert Carba-R surveillance result was positive, contact isolation was conducted immediately. Specimens of suspected infection sites were collected for CRO culture. If CRO was cultured from relevant specimens before admission to the ICU, contact isolation was conducted immediately after admission. Positive patients were isolated in a single room placement (one patient per room).

**Figure 1:**
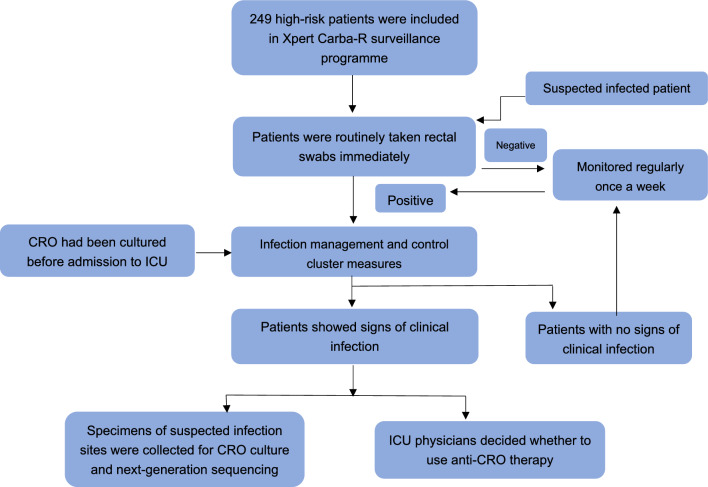
Active Xpert Carba-R surveillance programme.

CRO positive patients were managed according to the infection management and control cluster measures, and/or received corresponding intervention measures (such as anti-infection treatment based on the surveillance results). Moreover, negative high-risk patients required regular surveillance Specimens of suspected infection sites were immediately obtained for culture and next-generation sequencing. Antibiotic treatment was optimized according to bacterial culture and next-generation sequencing results. Regular surveillance was continued until the patient's infection was under control. The active surveillance program is illustrated in Fig. 1.

### Data collection and outcome assessment

The following data items were collected in the second period: sex, age, acute physiology and chronic health evaluation II (APACHE II), Sequential Organ Failure Assessment (SOFA), glucocorticoid use, history of diabetes, cardiovascular disease, cerebrovascular disease, blood disease, acquired immune deficiency syndrome, malignant tumor, chronic kidney disease, organ transplantation, gastrointestinal disease, hepatic and gall disease, autoimmune disease, treatment and procedures performed before infection (e.g., steroids, chemotherapy, and antibiotic therapy), culture, and surveillance results. The primary outcome was prevalence of CRO colonization and infection. Two estimates of CRO occurrence were calculated based on data collected by researchers from the hospital’s infection management system. The proportion of active surveillance patients with newly detected CRO was calculated by dividing the number of CRO-positive patients who screened positive by the total number of patients screened. The overall CRO prevalence was calculated by dividing the total number of CRO-positive patients (patients previously known to have CRO and Xpert Carba-R surveillance-positive patients or who were culture-positive) by the total number of ICU patients present during the study. The occurrence of CRO was defined as colonization, positive blood cultures, or positive cultures from other sterile sources. Positive cultures from respiratory, urine, and surgical wounds were defined as infections according to the Centers for Disease Control and Prevention/National Healthcare Safety Network criteria^15^.

### Infection management and control cluster measures

ICU staff paid particular attention to the nosocomial transmission of CRO and formulated cluster control measures in light of these guidelines. Patients in two groups (2020–2022) with CRO colonization and infection were managed according to the same bundles, including single-room isolation, contact isolation, enhanced environmental cleaning, scrubbing the skin daily with 2% chlorhexidine gluconate, and healthcare worker education and adherence monitoring, with a focus on hand hygiene. When the patients who later screening turned from positive to negative, they could be released from contact isolation after two consecutive negative tests. Meanwhile, they would be also screened regularly and at any time for suspicious infection. All patients who met the inclusion criteria were included in the study, and rectal swabs were collected and monitored over time. Researchers conducted quality control evaluations on the collected and monitored rectal specimens monthly and provided feedback on the research process, including the compliance rate of bundle components of measures.

### Statistical analysis

Continuous data are presented as mean ± standard deviation (SD) and median (interquartile range) according to data distribution, while the event (proportion) was applied for categorical data. The differences between groups for continuous data were assessed using an independent *t* test, and the mean difference with 95% confidence interval (CI) was calculated. The chi-squared test was then applied to assess between-group differences for categorical data, and odds ratios (OR) with 95% CI were calculated using multivariate logistic regression. All reported *P* values were two-sided, and the significance level was 0.05. All statistical analyses were performed using SPSS Statistics for Windows version 26.0 (IBM Corp., Armonk, NY, USA).

## Results

### CRO incidence and length of ICU stay

A total of 3765 patients were included in the two groups; baseline characteristics in the Xpert Carba-R surveillance and control groups are shown in Table 1. There were no significant between-group differences for sex, age, APACHE II, SOFA, glucocorticoid use, white blood cell, creatinine, serum Na^+^, serum K^+^, mechanical ventilation time, and chronic health status. Table 2 shows the CRO incidence and length of ICU stay in the Xpert Carba-R surveillance and control groups. CRO was detected in 205 of 1755 patients in the control group, demonstrating an overall CRO incidence of 11.68%, whereas 185 CRO cases were detected, with an overall CRO incidence of 9.2%. We noted that Xpert Carba-R surveillance was associated with a reduced CRO risk (*P* = 0.013), especially for CRAs + CRPA (*P* = 0.043), CRKP (*P* = 0.001), and CRE (*P* = 0.008) compared with that in the control group. However, there were no significant between-group differences for risk of CRAs (*P* = 0.140), CRPA *P* = 0.161), or CRE except CRKP (*P* = 0.259). Moreover, the difference in length of ICU stay between the Xpert Carba-R surveillance and control groups was not statistically significant (*P* = 0.743).

**Table 1:** Baseline characteristics of Xpert Carba-R active surveillance group and control group patients.

Variable	Xpert Carba-R Active surveillance (n = 2010)	Control group (n = 1755)	*P* value
Sex			
Male	1242 (61.8%)	1114 (63.5%)	0.287
Age (years)	60.60 ± 15.944	60.91 ± 15.257	0.553
APACHE II	17.74 ± 6.575	18.09 ± 5.883	0.348
SOFA	5.48 ± 3.574	5.35 ± 3.290	0.272
GCS	10.23 ± 4.931	9.98 ± 4.289	0.096
White blood cell (*10^9/L)	11.03 ± 6.62397	10.95 ± 6.53611	0.709
Creatinine (umol/l)	126.08 ± 154.2983	121.18 ± 111.0437	0.270
Serum Na + (mmol/l)	140.73 ± 7.9656	140.75 ± 8.5972	0.924
Serum K + (mmol/l)	3.82 ± 1.0039	3.78 ± 0.8811	0.257
Mechanical ventilation time (h)	65.31 ± 154.592	61.91 ± 189.217	0.544
Chronic health status	225(11.19%)	224(12.76%)	0.140

Chronic health status: combined with chronic organ dysfunction and immunosuppression.

**Table 2:** Incidence of CRO and Length of ICU stay.

Outcome	Xpert Carba-R Active surveillance group (n = 2010)	Control group (n = 1755)	OR and 95% CI	*P* value
CRO	185 (9.2%)	205 (11.68%)	0.77 (0.62–0.95)	0.013
CRAs	114 (5.67%)	120 (6.83%)	0.82 (0.63–1.07)	0.140
CRPA	36 (1.79%)	43 (2.45%)	0.73 (0.46–1.14)	0.161
CRAs + CRPA	150 (7.46%)	163 (9.28%)	0.79 (0.62–0.99)	0.043
CRKP	55 (2.73%)	84 (4.78%)	0.56 (0.40–0.79)	0.001
CRE except CRKP	15 (0.75%)	8 (0.46%)	1.64 (0.69–3.88)	0.259
CRE	70 (3.48%)	92 (5.24%)	0.65 (0.47–0.90)	0.008
Incidence of CRO in High-risk patients	87/249(34.9%)	96/218(44.0%)		
Length of ICU stay	4.67 ± 6.31	4.75 ± 8.36	− 0.08 (− 0.56 to 0.40)	0.743

*CRA* carbapenem-resistant Acinetobacter, *CRO* carbapenem-resistant microorganism, *CRPA* carbapenem-resistant Pseudomonas aeruginosa, *CRKP* carbapenem resistant Klebsiella pneumoniae, *CRE* Carbapenem resistant enterobacteriaceae, *ICU* intensive care unit.

**Figure 2:**
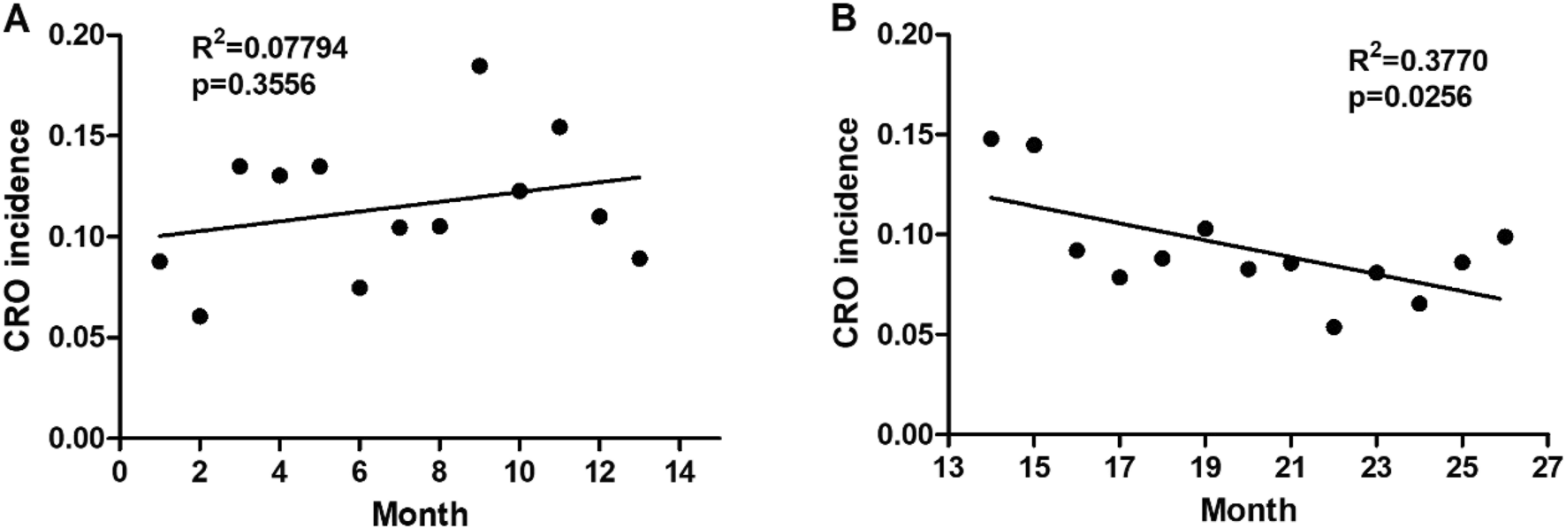
The trend of the CRO incidence. A significant decrease in the monthly incidence of CRO found by linear regression.

Regression analysis showed that there was no significant change of CRO incidence in monthly trend in control group (p = 0.3556), but CRO incidence showed a decreasing trend in active surveillance group (p = 0.0256) (Fig. 2).

### Time between ICU admission and positive detection of CRO

Among 249 high-risk patients in the Xpert Carba-R surveillance group, 38 manifested the presence of carbapenem resistance genes. Among the 38 surveillance positive patients, 30 patients demonstrated CRO-positive culture. The remaining 211 patients did not been detected carbapenem resistance genes, but 49 patients demonstrated CRO positive culture (Table 3). There were significant differences between the control group and Xpert Carba-R surveillance positive patients with regard to time between ICU admission and CRO positive results (*P* = 0.005). Meanwhile, time to obtain positive culture results was significantly longer than the time to obtain active surveillance results (*P* = 0.010). However, there was no significant difference in the time to obtain results between the control group and “Xpert Carba-R surveillance and cultured positive patients” (*P* = 0.630).

**Table 3:** Time between ICU admission and CRO positive results.

	Group		MD and 95%CI	*P* value
Time between ICU admission and CRO positive results, days	Control group(n = 1755)	Xpert Carba-R Active surveillance positive(n = 38)		
	7.97 ± 7.36	4.54 ± 3.39	3.43 (1.06 to 5.80)	0.005
	Control group(n = 1755)	Xpert Carba-RActive surveillance positive and cultured positive (n = 30)		
	7.97 ± 7.36	7.30 ± 5.27	0.67(-2.08 to 3.42)	0.630
	Xpert Carba-RActive surveillance positive(n = 38)	Xpert Carba-RActive surveillance positive and cultured positive(n = 30)		
	4.54 ± 3.39	7.30 ± 5.27	2.76(0.67 to 4.85)	0.010
	Xpert Carba-RActive surveillance negative and cultured positive (n = 49)	Xpert Carba-RActive surveillance positive (n = 38)		
	7.62 ± 3.06	4.54 ± 3.39	3.08 (1.93 to 4.23)	< 0.001

### Patients at high risk for CRO

A total of 249 high-risk patients were included in the active surveillance program, and baseline characteristics of patients in the CRO-positive and CRO-negative groups are shown in Supplementary S2. Most characteristics showed no difference between the CRO-positive and CRO-negative groups, while there was a significant difference for prior blood disease (*P* = 0.013). Moreover, the positive rate of resistance genes was 15.2% (38/249), with positive rates of KPC, NDM, IMP, OXA-48, and VIM of 10.04%, 4.8%, 2.4%, 0.4%, and 0.4%, respectively (Table 4). Among 38 patients with carbapenem resistance genes, 30 had cultured specimens that routinely exhibited CRO; the consistency with Xpert Carba-R surveillance result was 78.90%. Among Xpert Carba-R surveillance -negative patients, 49 (23.2%) patients had CRO-positive cultures. There were significant differences between the CRO-negative and CRO-positive groups (*P* < 0.001). In Xpert Carba-R surveillance-positive patients, there were 20 cases of CRKP, seven of CRPA, eight of CRAs, and one of CRE (except CRKP) that were identified using conventional culture. Other pathogens (not CRO) were cultured in six patients and were negative in two patients. In Xpert Carba-R surveillance-negative patients, there were 37 cases of CRAs, 10 of CRKP, 4 of CRKP, and 3 of CRE (excluding CRKP) that were identified using conventional culture. There were significant differences between CRO-negative and CRO-positive groups with regard to microorganism-positive culture (*P* < 0.001). Furthermore, there were significant differences between the CRO-negative and CRO-positive groups in the time between ICU admission and CRO-positive results (*P* < 0.001) and length of hospital stay before surveillance (*P* = 0.002). Finally, there were significant between-group differences for carbapenem antibiotic use 90 days before surveillance (*P* = 0.001), corticosteroid use 90 days before surveillance (*P* = 0.028), and surgery 90 days before surveillance (*P* = 0.003), while no significant difference was observed between groups for enteral nutrition before surveillance (*P* = 0.901) (Supplementary S3).

**Table 4:** The carbapenem resistance genes distribution for high risk patients.

The resistant genes distribution	Carbapenem resistance genes positive rate
All	15.2% (38/249)
KPC	10.04% (25/249)
NDM	4.8% (12/249)
IMP	2.4% (6/249)
OXA-48	0.4% (1/249)
VIM	0.4% (1/249)

*IMP* imipenemase, *KPC* klebsiella pneumoniae carbapenemase, *NDM* New Delhi metallo-β-Lactamase, *OXA-48* oxacillinase-48, *VIM* verona integron-mediated metallo-β-lactamase.

## Discussion

The current study reported CRO infection rates and trends in ICU patients and assessed the role of Xpert Carba-R surveillance of CRO by Xpert Carba-R on CRO incidence in critically ill patients. A total of 3765 patients were enrolled, and characteristics of Xpert Carba-R surveillance and control groups in high-risk patients were well balanced. The CRO incidence was 11.68% and 9.2% of patients in the two groups, respectively, and individualized active surveillance of carbapenem resistance genes by Xpert Carba-R was associated with a reduced CRO risk, especially for CRAs + CRPA, CRKP, and CRE. Meanwhile, we found that active surveillance using Xpert Carba-R shortened the time to CRO detection. Moreover, Xpert Carba-R surveillance-positive and-negative high-risk patients significantly differed with respect to CRO-positive culture, microorganism-positive culture, time between ICU admission and CRO positivity, length of hospital stay before surveillance, use of carbapenem antibiotics in the 90 days prior to surveillance, corticosteroid use in the 90 days before surveillance, and surgery in the 90 days before surveillance.

Our study found that CRO incidence was 11.68% and 9.2% (4.78% and 2.73% for CRKP, 5.24% and 3.48% for CRE, 6.83% and 5.67% for CRAs, and 2.45% and 1.79% for CRPA) in the active surveillance-positive and -negative groups, respectively. Several studies have already addressed the epidemiology and incidence of CRE and CRAs^6,10,16–20^, while one study has reported the drug resistance phenotype and molecular epidemiological characteristics of CPKP in children^21^. Our results demonstrate that early Xpert Carba-R surveillance of carbapenem resistance genes is associated with reduction of the incidence of CRO in the ICU and should provide support for other healthcare facilities that are working to reduce the burden of CRO in their patient populations; these findings are consistent with those of previous studies^22,23^. However, the length of hospital stay was not significantly different between the two groups; this may be explained by the effect of patient characteristics, disease status, and treatment regimens on length of hospital stay.

Studies have shown that the CRO carrier rate in hospitalized patients is low, suggesting that because of the relatively low prevalence of CRO, it is not cost-effective to screen all ICU admissions^4,24,25^. High-risk individualized active surveillance in high-risk settings, such as the ICU, helps to detect asymptomatic CRO carriers who can serve as reservoirs for transmission during hospitalization. Moreover, the use of active surveillance for high-risk patients may be more targeted, which may reduce the consumption of medical resources^22,26^. In our study, patients at high risk of CRO infection were selected, and 249 high-risk patients were enrolled in the second period, 38 of whom exhibited the presence of carbapenem-resistant genes and 49 of whom were Xpert Carba-R surveillance-negative but who demonstrated a CRO-positive culture. The positive detection rate of CRO in high-risk patients was 34.94% (87/249), which was much higher than the rate of CRO in hospitalized patients reported previously, including our department. The results showed that Xpert Carba-R surveillance of CRO in high-risk groups is feasible and efficient. The potential reason why 49 patients showed negative results for Xpert Carba-R surveillance but who had CRO-positive cultures may be that these patients did not have rectal colonization of CRO, while other infection statuses were observed. Five carbapenem resistance genes were detected (KPC, 25; NDM, 12; IMP, 6; OXA-48, 1; and VIM, 1). In the future, clinicians may be able to select antibiotics based on the type of carbapenem resistance gene present.

Our study found a 78.90% consistency of CRO positivity between Xpert Carba-R surveillance and routine culture. This suggests that Xpert Carba-R surveillance-positive patients are likely to develop corresponding pathogens that are culture-positive and even become potentially infected later. Moreover, the incidence of CRO-positive culture was 23.30% in Xpert Carba-R surveillance-negative patients, which was significantly lower than that in Xpert Carba-R surveillance-positive patients (78.90%). The reasons for CRO-positive culture in Xpert Carba-R surveillance-negative patients may be explained by subsequent infection in the ICU, wheras the Xpert Carba-R mainly focused on patients carrying multidrug-resistant bacteria; thus, false negative results for CRO indicated by Xpert Carba-R should be cautiously monitored. The time between ICU admission and CRO-positive (active surveillance or culture) results was 7.62 and 4.54 days in the Xpert Carba-R surveillance-positive and-negative groups, respectively. There was an average difference of 3 days between the positive results of Xpert Carba-R surveillance and conventional culture, indicating that Xpert Carba-R surveillance can obtain results more quickly to guide the prevention and control of hospital infection. Therefore, hospital staff can react in advance according to the surveillance results and obtain potential benefits such as reducing transmission and guiding the application of antibiotics.

Several limitations of this study should be acknowledged. First, this study was designed as a single-center, before-after study, and the sample size was small. Second, background therapies for critically ill patients could affect CRO progression, which should be adjusted for in multivariate analysis. Finally, Xpert Carba-R surveillance only for high-risk patients admitted to the ICU and the epidemiology of carbapenem resistance might have been overestimated.

## Supplementary Information

Supplementary Figure 1.

Supplementary Table 1.

Supplementary Table 2.

## Data Availability

The datasets used and/or analysed during the current study available from the corresponding author on reasonable request.

## Abbreviations

APACHE II: Acute physiology and chronic health evaluation II
CI: Confidence interval
CRAs: Carbapenem-resistant acinetobacter
CRE: Carbapenem-resistant Enterobacteriaceae
CRO: Carbapenem-resistant microorganisms
CRPA: Carbapenem-resistant *Pseudomonas aeruginosa*
ICU: Intensive care unit
IMP: Imipenemase
KPC: *Klebsiella pneumoniae* Carbapenemase
NDM: New Delhi metallo-β-lactamase
OR: Odds ratio
OXA: Oxacillinase
SOFA: Sequential organ failure assessment
VIM: Verona integron-mediated metallo-β-lactamase
