# Supplementary material for: Individualized active surveillance for carbapenem-resistant microorganisms using Xpert Carba-R in intensive care units
Source: Sci Rep. 2023 Jun 12;13:9527. doi: 10.1038/s41598-023-36321-y (PMC10261131; doi:10.1038/s41598-023-36321-y)
Supplement: Supplementary file 1 — Supplementary Figure 1. [file 41598_2023_36321_MOESM1_ESM.docx]

**Figure 1.** **Study Protocol**

Xpert Carba-R surveillance group

249 high-risk patients were included in Xpert Carba-R surveillance programme

Control group

Retrospectively studied the clinical data of all 1755 patients admitted in the ICU from March 2020 to March 2021

2010 patients during April 2021 to April 2022 were assessed for eligibility

Outcome indicators of two groups were statistically analyzed

790 expected length of ICU stay less than 1 day

473 did not meet inclusion criteria

332 had been enrolled in another trial 106 refused to undergo rectal swab

60 other excluded
