# Supplementary material for: Individualized active surveillance for carbapenem-resistant microorganisms using Xpert Carba-R in intensive care units
Source: Sci Rep. 2023 Jun 12;13:9527. doi: 10.1038/s41598-023-36321-y (PMC10261131; doi:10.1038/s41598-023-36321-y)
Supplement: Supplementary file 2 — Supplementary Table 1. [file 41598_2023_36321_MOESM2_ESM.docx]

Supplementary 2. Baseline characteristics of high-risk patients

| Variable | CRO negative (n=211) | CRO positive (n=38) | *P* value |
| --- | --- | --- | --- |
| Sex |  |  | 0.820 |
| Male | 146(69.2%) | 27(71.1%) |  |
| Age (years) | 61.2 | 59.3 | 0.490 |
| APACHE II | 19.34 | 21.62 | 0.067 |
| SOFA | 8.50 | 8.22 | 0.680 |
| GCS | 10.47 | 9.42 | 0.095 |
| History of disease |  |  |  |
| Diabetes | 34 | 11 | 0.058 |
| Cardiovascular disease | 89 | 12 | 0.220 |
| Cerebrovascular Disease | 41 | 8 | 0.810 |
| Blood disease | 1 | 2 | 0.013 |
| AIDS | 3 | 0 | 0.460 |
| Malignant tumor | 43 | 6 | 0.512 |
| Chronic kidney disease | 15 | 2 | 0.670 |
| Organ transplantation | 4 | 1 | 0.760 |
| Gastrointestinal disease | 22 | 2 | 0.320 |
| Hepatic and gall diseases | 21 | 1 | 0.140 |
| Autoimmune Disease | 2 | 0 | 0.540 |

*AIDS: acquired Immune Deficiency Syndrome; APACHE II: acute physiology and chronic health evaluation II; CRO: carbapenem-resistant microorganism; GCS: glucocorticoid; SOFA: sequential organ failure assessment
