# Supplementary material for: Individualized active surveillance for carbapenem-resistant microorganisms using Xpert Carba-R in intensive care units
Source: Sci Rep. 2023 Jun 12;13:9527. doi: 10.1038/s41598-023-36321-y (PMC10261131; doi:10.1038/s41598-023-36321-y)
Supplement: Supplementary file 3 — Supplementary Table 2. [file 41598_2023_36321_MOESM3_ESM.docx]

Supplementary 3. The characteristics according to Xpert Carba-R surveillance status

| Outcome | Xpert Carba-R surveillance negative (n=211) | Xpert Carba-R surveillance positive (n=38) | OR and 95%CI | *P* value |
| --- | --- | --- | --- | --- |
| CRO Cultured positive | 49 (23.30%) | 30 (78.90%) | 0.08 (0.03-0.19) | < 0.001 |
| Microorganism Cultured positive | 78 (36.90%) | 36 (94.74%) | 0.03 (0.01-0.14) | < 0.001 |
| Time between ICU admission and CRO positive results, days | 7.62±3.06 | 4.54±3.39 | 3.08 (1.93 to 4.23) | < 0.001 |
| Length of hospital stay before surveillance | 8.76±6.88 | 22.87±28.56 | -14.11 (-23.24 to -4.98) | 0.002 |
| Carbapenem antibiotics use in past 90 days before surveillance | 25 (11.85%) | 13 (34.21%) | 0.26 (0.12-0.57) | 0.001 |
| Corticosteroid use in past 90 days before surveillance | 30 (14.22%) | 11 (28.95%) | 0.41 (0.18-0.91) | 0.028 |
| Surgery in the past 90 days before surveillance | 77 (36.49%) | 24 (63.16%) | 0.34 (0.16-0.69) | 0.003 |
| Enteral Nutrition before surveillance | 141 (66.82%) | 25 (65.79%) | 1.05 (0.51-2.17) | 0.901 |

*CI: confidence interval; CRO: carbapenem-resistant microorganism; ICU: intensive care unit; MD: mean difference; OR: odds ratio.
